# Metal-Resistant PGPR Strain *Azospirillum brasilense* EMCC1454 Enhances Growth and Chromium Stress Tolerance of Chickpea (*Cicer arietinum* L.) by Modulating Redox Potential, Osmolytes, Antioxidants, and Stress-Related Gene Expression

**DOI:** 10.3390/plants12112110

**Published:** 2023-05-26

**Authors:** Enas M. El-Ballat, Sobhy E. Elsilk, Hayssam M. Ali, Hamada E. Ali, Christophe Hano, Mohamed A. El-Esawi

**Affiliations:** 1 Botany Department, Faculty of Science, Tanta University, Tanta 31527, Egypt; enas.elballat@science.tanta.edu.eg (E.M.E.-B.); sobhy.elsilk@science.tanta.edu.eg (S.E.E.); 2 Department of Botany and Microbiology, College of Science, King Saud University, Riyadh 11451, Saudi Arabia; hayhassan@ksu.edu.sa; 3 Department of Biology, College of Science, Sultan Qaboos University, Muscat 123, Oman; helsayedali@gmail.com; 4 Botany and Microbiology Department, Faculty of Science, Suez Canal University, Ismailia 41522, Egypt; 5 Laboratoire de Biologie des Ligneux et des Grandes Cultures, INRAE USC1328, Campus Eure et Loir, Orleans University, 45067 Orleans, France; hano@univ-orleans.fr; 6 Photobiology Research Group, Sorbonne Université CNRS, 75005 Paris, France

**Keywords:** *Azospirillum brasilense* EMCC1454, chromium tolerance, osmolytes, antioxidant machinery, stress-related gene expression, chickpea

## Abstract

Heavy metal stress, including from chromium, has detrimental effects on crop growth and yields worldwide. Plant growth-promoting rhizobacteria (PGPR) have demonstrated great efficiency in mitigating these adverse effects. The present study investigated the potential of the PGPR strain *Azospirillum brasilense* EMCC1454 as a useful bio-inoculant for boosting the growth, performance and chromium stress tolerance of chickpea (*Cicer arietinum* L.) plants exposed to varying levels of chromium stress (0, 130 and 260 µM K_2_Cr_2_O_7_). The results revealed that *A. brasilense* EMCC1454 could tolerate chromium stress up to 260 µM and exhibited various plant growth-promoting (PGP) activities, including nitrogen fixation, phosphate solubilization, and generation of siderophore, trehalose, exopolysaccharide, ACC deaminase, indole acetic acid, and hydrolytic enzymes. Chromium stress doses induced the formation of PGP substances and antioxidants in *A. brasilense* EMCC1454. In addition, plant growth experiments showed that chromium stress significantly inhibited the growth, minerals acquisition, leaf relative water content, biosynthesis of photosynthetic pigments, gas exchange traits, and levels of phenolics and flavonoids of chickpea plants. Contrarily, it increased the concentrations of proline, glycine betaine, soluble sugars, proteins, oxidative stress markers, and enzymatic (CAT, APX, SOD, and POD) and non-enzymatic (ascorbic acid and glutathione) antioxidants in plants. On the other hand, *A. brasilense* EMCC1454 application alleviated oxidative stress markers and significantly boosted the growth traits, gas exchange characteristics, nutrient acquisition, osmolyte formation, and enzymatic and non-enzymatic antioxidants in chromium-stressed plants. Moreover, this bacterial inoculation upregulated the expression of genes related to stress tolerance (*CAT*, *SOD*, *APX*, *CHS*, *DREB2A*, *CHI,* and *PAL*). Overall, the current study demonstrated the effectiveness of *A. brasilense* EMCC1454 in enhancing plant growth and mitigating chromium toxicity impacts on chickpea plants grown under chromium stress circumstances by modulating the antioxidant machinery, photosynthesis, osmolyte production, and stress-related gene expression.

## 1. Introduction

Heavy metals can be accumulated in plant tissues and organs, causing adverse impacts on the performance and yields of crops [1,2,3]. One of the major heavy metals being toxic to plants is chromium (Cr), which can be found in soil and water [4], and can enter the soil initially from natural sources such as chromite. Excess Cr found in soil and water is due to several human activities, including tanning, mining, and other activities that produce Cr-containing effluents and atmospheric depositions [3,5,6]. Like other heavy metals, Cr can have detrimental impacts on plant growth, morphology, performance, and gene expression [3,7,8,9]. With respect to growth and developmental processes, it is indicated that Cr excess in plants influences the germination rate, roots and shoots growth rates, roots and shoots biomass, chlorophyll content, nutrient uptake, nitrogen metabolism, enzyme activity, and overall yield [3,10,11,12]. Furthermore, Cr toxicity restricts plant photosynthesis and water use efficiency, alters the balance of hormones in plants, and causes accumulation of reactive oxygen species (ROS) and oxidative stress [6,13,14]. In terms of gene expression, Cr toxicity regulates the expression of genes mediating plant stress responsiveness [15]. To combat these Cr toxicity impacts, plants have evolved different strategies such as formation of solutes and osmolytes as well as induction of ROS-scavenging antioxidants and key genes involved in phenylpropanoid biosynthetic pathways [16,17,18,19].

Diverse approaches have been adopted to reverse the impact of heavy metals toxicity on plants. Among these strategies, the implementation of beneficial soil microorganisms is an environmentally friendly efficient and sustainable technology. Earlier studies demonstrated the crucial roles of plant growth-promoting rhizobacteria (PGPR) in boosting performance of crops and their resistance to heavy metal stress via interacting with plants, secreting phytohormones, and mediating different physiobiochemical and molecular mechanisms [1,20,21,22,23]. These mechanisms include induction of free radical scavenging systems, alteration of root architecture, and biosynthesis of osmolytes [24,25]. *Azospirillum* species are among the most beneficial PGPR found in rhizospheres of cereals and grasses and are known to colonize the plant root, fix nitrogen, secrete auxins, solubilize phosphate, modulate antioxidant defense systems, and consequently boost plants growth and mitigate the effects of environmental stress circumstances [22,25,26,27,28]. *Azospirillum lipoferum* revealed great potential to significantly mitigate the negative impacts of salinity stress by enhancing the development and performance of wheat and chickpea [25,29]. Moreover, inoculation of *A. brasilense* strain Cd (ATCC 29729) reduced the damaging effects of saline water on chickpea performance and yield [30]. It is worth mentioning that the use of *Azospirillum* species for enhancing plants’ tolerance to Cr toxicity is still an area of ongoing research, and more studies are required to dissect the underlying mechanisms and to optimize the conditions for application.

Chickpea (*Cicer arietinum* L.) is an economically staple crop cultivated globally, and represents a valuable source of carbohydrates, proteins, fats, vitamin, and nutrients [31,32]. Chickpea crops experience several stresses, such as from salt and heavy metals, which restrict their performance and yield [33,34]. Syed et al. [34] demonstrated the negative consequences of cadmium stress on chickpea performance, which was then promoted by *Pseudomonas fluorescens* PGPR-7 inoculation. Adhikary et al. [35] also demonstrated that *Pseudomonas citronellolis* boosted chickpea performance and dry biomass under arsenic stress. Considering the potentiality of PGPR in inducing resistance to heavy metals, seeking new metal-tolerant PGPR strains that could boost the growth and Cr stress resistance of chickpea crop is very much needed. Moreover, much less is known about the potential role of *A. brasilense* in alleviating Cr toxicity affecting chickpea. Therefore, the current study evaluated the potential of *A. brasilense* strain EMCC1454 as a significant bio-inoculant for boosting chickpea growth and tolerance to Cr toxicity. It elucidated the diverse underlying mechanisms that *A. brasilense* EMCC1454 triggers in response to Cr stress in chickpea. The morpho–physiobiochemical and molecular approaches have been assessed.

## 2. Materials and Methods

### 2.1. Bacterial Strain and Evaluating its Cr Stress Tolerance

The bacterial strain *Azospirillum brasilense* EMCC1454 used in the current investigation was from the Microbiological Resources Centre located at the Faculty of Agriculture, Ain Shams University, Egypt. The Cr stress tolerance of this bacterial strain was tested by incubating the bacterium in a nutrient broth amended with varying levels of K_2_Cr_2_O_7_ (0, 130, 260, and 400 µM) for 0, 12, 24, 48, and 72 h at 28 °C. Bacterial growth was then evaluated by monitoring the optical density (OD) at 600 nm. This experiment was performed three times.

### 2.2. Examination of Plant Growth-Promoting Characteristics of A. brasilense EMCC1454

#### 2.2.1. Nitrogen Fixation Ability

The nitrogen-fixing potential of *A. brasilense* EMCC1454 was assayed using the protocol reported previously by Weselowski et al. [36]. The bacterium was cultivated on a nitrogen-free malate agar medium, followed by incubation for 5 days at 28 °C. Growth of bacterial colonies on the nitrogen-free minimal agar was an indication of the bacterium’s capacity to fix nitrogen.

#### 2.2.2. Siderophore Formation

The siderophore formation of *A. brasilense* EMCC1454 was evaluated using chrome azurol S (CAS) agar medium according to the protocol outlined by Schwyn and Neilands [37]. In brief, freshly grown bacterial culture was streaked on CAS agar and then incubated at 28 °C for 72 h. The formation of orange–yellow halo zone surrounding colonies indicated siderophore formation.

#### 2.2.3. Phosphate Solubilization Potential

The potential of *A. brasilense* EMCC1454 to solubilize phosphate was assessed by culturing it on Pikovskaya’s agar medium (0.05% yeast extract, 1% glucose, 0.02% KCl, 0.05% (NH_4_)_2_SO_4_, 0.01% MgSO_4_·_7_H_2_O, 0.0005% MnSO_4_, 0.0005% FeSO_4_, 0.1% Ca_3_HPO_4_, 0.2% calcium phytate, and 1.5% agar), followed by incubation for two weeks at a temperature of 28 °C. Following the incubation time, the appearance of a transparent halo zone surrounding colonies on the agar medium was an indication of the capability of bacteria to solubilize phosphate.

#### 2.2.4. Hydrolytic Enzyme Activities

Nutrient broth was used to culture the bacterium *A. brasilense* EMCC1454 for 24 h at 28 °C. The bacterial culture was utilized for determining the activities of hydrolytic enzymes (amylase, protease, chitinase, and cellulase enzymes) on agar plate methods. To assess amylase production, the bacterial culture was introduced to a plate of nutrient agar that contained 0.2% soluble starch. Subsequently, Lugol’s iodine was applied to the culture grown. The formation of clear halo zones surrounding colonies indicated amylase production [38]. Furthermore, the ability of the bacterial culture to secret protease was detected by streaking it onto a plate containing milk agar medium, which was then incubated at 30 °C for 24 h. The development of a clear halo zone surrounding the colonies indicated the ability of the bacterial cells to produce protease [39]. The chitinase production by the bacterial culture was also tested by streaking it on chitin agar medium, which was then incubated for 3–7 days at 30 °C. A positive result for chitinase production was indicated by the formation of a clear halo zone around bacterial colonies [40]. Additionally, to test the bacterium cellulase activity, 5 µL of each bacterium was inoculated onto CMC agar, as outlined by Gohel et al. [41].

### 2.3. Assessing the Capability of A. brasilense EMCC1454 to Secrete Acid Compounds

The acid compounds-producing potential of *A. brasilense* EMCC1454 was evaluated by monitoring the pH of its bacterial suspension at 24 h intervals. In brief, 1 mL of *A. brasilense* EMCC1454 suspended in nutrient broth was introduced to a growth medium comprising tryptone (10 g/L), glucose (10 g/L), and NaCl (5 g/L). The bacterial suspension pH value was monitored every 24 h. This experiment was repeated 5 times.

### 2.4. Assessing Cr Levels on Stress Tolerance Characteristics of A. brasilense EMCC1454

#### 2.4.1. Survivability of *A. brasilense* EMCC1454 under Cr Stress Conditions

The ability of *A. brasilense* EMCC1454 to survive at varying Cr stress levels was evaluated. Approximately, 25 μL of bacterial suspension was streaked into nutrient broth comprising varying Cr levels (0, 130, and 260 μM K_2_Cr_2_O_7_) and kept under shaking for 24 h. The bacterial population was then determined utilizing serial dilutions and the total plate count method [42].

#### 2.4.2. Trehalose Content of *A. brasilense* EMCC1454 under Cr Stress Conditions

Trehalose content of *A. brasilense* EMCC1454 under normal and Cr stress conditions was determined according to the procedures outlined by Rodríguez-Salazar et al. [43]. In brief, the bacterial isolate was cultured into Nfb liquid medium containing NH_4_^+^ at 30 °C for 24 h. The grown culture was subjected to centrifugation, washing, and resuspension in ethanol. Following incubation for 15 min at 85 °C, to recover the supernatant, the bacterial cells were exposed to centrifugation for 6 min at 12,000 rpm. Samples were then resuspended in distilled water and subjected to HPLC analysis.

#### 2.4.3. ACC (1-Aminocyclopropane-1-Carboxylic Acid) Deaminase Activity of *A. brasilense* EMCC1454 under Cr Stress Conditions

ACC deaminase activity of *A. brasilense* EMCC1454 was assessed under varying Cr levels (0, 130, and 260 μM K_2_Cr_2_O_7_) by assessing the concentration of α-ketobutyrate formed by ACC degradation using ACC deaminase enzyme [44]. α-ketobutyrate content was quantified by monitoring the sample optical density at 540 nm, followed by a comparison to standard curves created using various concentrations of α-ketobutyrate solution (0.1 to 1.0 µmol).

#### 2.4.4. Indole Acetic Acid (IAA) Production by *A. brasilense* EMCC1454 under Cr Stress Conditions

The procedure outlined by Chen et al. [45] was utilized to quantify the amount of IAA produced by *A. brasilense* EMCC1454 grown at varying Cr levels (0, 130, and 260 μM K_2_Cr_2_O_7_). *A. brasilense* EMCC1454 was cultured into tryptic soy broth medium amended with 200 µg mL^−1^ tryptophan and incubated for 3 days at 28 °C. A tryptic soy broth lacking tryptophan served as a control. The samples were subjected to centrifugation at 5500× *g* for 12 min, and 1 mL of supernatant was combined with 4 mL of Salkowski reagent (1 mL of 0.5 M FeCl_3_ solution and 50 mL of 35% perchloric acid) and 0.1 mL of orthophosphoric acid. The mixed solutions were then kept in the dark for 25 min, and the content of IAA was assessed by monitoring the absorbance at 530 nm and using a standard curve of pure IAA solution (10–100 µg/mL).

#### 2.4.5. Exopolysaccharide (EPS) Production by *A. brasilense* EMCC1454 under Cr Stress Conditions

A modified Congo red binding test [46] was applied to determine the EPS production by *A. brasilense* EMCC1454 grown at varying Cr levels (0, 130, and 260 μM K_2_Cr_2_O_7_). To perform this test, the bacterial cultures were first vortexed followed by centrifugation for 12 min at 13,000× *g*. Pellets were added into 1% tryptone (300 µL) mixed with 12 μg mL^−1^ Congo red, and left in incubator with shaking for 2 h at 37 °C. After another round of centrifugation, the absorbance of Congo red-containing supernatant was monitored at 490 nm. An uninoculated culture medium representing a control was utilized.

#### 2.4.6. Antioxidant Enzyme Activities of *A. brasilense* EMCC1454 under Cr Stress Conditions

Antioxidant enzyme assays were performed for the extracts of control and Cr stress-treated *Azospirillum* cells. The samples were set up following the method outlined previously by Mukherjee and Choudhuri [47]. In brief, the sample (0.5 gm) was ground into a fine powder. The resulting powder was mixed with 10 mL of 100 mM phosphate buffer (KH_2_PO_4_/K_2_HPO_4_, pH 7.0), along with 0.1 gm polyvinylpyrrolidone and 0.1 mM Na_2_EDTA. The mixture was subjected to homogenization, filtration, and centrifugation at 13,000 rpm for 12 min at 4 °C. Supernatant was then utilized to measure antioxidant enzyme assay.

To estimate the level of ascorbate peroxidase (APX), a reduction in ascorbate optical density was recorded at 290 nm according to the procedure outlined by Chen and Asada [48]. The reaction was performed in a 2 mL extract consisting of 0.4 mM ascorbate, 50 mM phosphate buffer (pH 7.0), and 0.4 mM H_2_O_2_. Catalase enzyme activity (CAT) was measured following the methodology documented by Aebi [49] with a slight modification. In brief, a cell-free extract (0.5 mL) was combined with 1 mL of a reaction solution comprising 30% (w/v) H_2_O_2_ and 50 mM phosphate buffer (pH 7.0). A reduction in absorbance was monitored at 240 nm to determine CAT activity.

To determine peroxidase (POD) activity, guaiacol was utilized as a substrate. The reaction solution was made up of enzyme extract, 20 mM guaiacol, 10 mM KH_2_PO_4_/K_2_HPO_4_ (pH 7.0), and 10 mM H_2_O_2_. The spectrophotometric measurement of the increase in absorbance at 470 nm indicated the generation of tetraguaiacol. To determine superoxide dismutase (SOD) level, the protocol outlined by Dhindsa et al. [50] was followed. The reaction solution contained 0.5 mL enzyme extract, 50 mM phosphate buffer (pH 7.8), 0.025 mM *ρ*-nitro blue tetrazolium chloride (NBT), 50 mM sodium bicarbonate, 0.1 mM EDTA, and 13 mM methionine. The reaction was initiated through addition of 0.002 mM riboflavin. The tubes were subjected to two 15W fluorescent lamps for 15 min. The reaction was then terminated by turning off the light and wrapping the test tubes with a black cloth. The absorbance was recorded at 560 nm to determine SOD activity. 

#### 2.4.7. Antioxidant Capacity of *A. brasilense* EMCC1454 under Cr Stress Conditions

Antioxidant capacity of the extracts of control and Cr stress-treated *Azospirillum* cells was determined by β-carotene-linoleic acid, 2,2-diphenyl-1-picrylhydrazyl (DPPH), and superoxide-scavenging (SOS) analyses. β-Carotene-linoleic acid and DPPH analyses were performed as previously reported by El-Esawi et al. [51]. β-Carotene-linoleic acid and DPPH results were represented as IC_50_ in μg mL^−1^. SOS analysis was also performed following the procedures outlined by Srinivasan et al. [52].

### 2.5. Pot Experiment and Chickpea Growth

*Azospirillum brasilense* strain EMCC1454 was incubated in nutrient broth at 28 °C for 24 h. Cells were then collected via centrifugation for 6 min at 2000× *g*. Pellets were resuspended in sterile water. A bacterial suspension of a concentration of 10^8^ colony-forming units (CFU) mL^−1^ was then adjusted and used to treat chickpea seedlings.

Chickpea (*Cicer arietinum* L., cv. Giza 3) seeds were treated with NaOCl (10%, *v*/*v*) for 5 min, followed by washing few times with distilled H_2_O, and then growing at 23 °C for 7 days. The healthy homogeneous seedlings were treated with *A. brasilense* EMCC1454 suspension for 25 min and transplanted into pots containing autoclaved substrates of sand, peat, and perlite (1:1:1, *v*/*v*/*v*). A fresh nutrient broth was used to treat the control plants for 25 min. The experimental design involved 6 treatments as follows: (i) plants not inoculated with Cr nor bacterial suspension (T1); (ii) plants treated with *A. brasilense* EMCC1454 (T2); (iii) plants inoculated with 130 µM K_2_Cr_2_O_7_ (T3); (iv) plants inoculated with 130 µM K_2_Cr_2_O_7_ and *A. brasilense* EMCC1454 (T4); (v) plants treated with 260 µM K_2_Cr_2_O_7_ (T5); and (vi) plants inoculated with 260 µM K_2_Cr_2_O_7_ and *A. brasilense* EMCC1454 (T6). Irrigation of chickpea plants was performed two times per week with a Hoagland solution comprising 0, 130, or 260 µM K_2_Cr_2_O_7_. Pots were arranged in a randomized complete block design in a growth chamber with growth conditions of 26/18 °C (day/night) and humidity of 76%. After 65 days, plants were harvested and used for further analysis.

### 2.6. Determination of Plant Morphological Characteristics

The harvested plant samples were cleaned by washing with sterile water. The length of both the shoots and roots was recorded. Subsequently, the roots and shoots were left to dry at 72 °C for 48 h to calculate their dry weight.

### 2.7. Estimation of Mineral Content in Plant Leaves

Approximately, 0.2 g of dried leafy powder was combined with 5 mL of H_2_SO_4_ in a homogenizer subjected to 200 °C for 4 h. H_2_O_2_ was then introduced into the mixture and left to react in a digester for 1 h. The solution was filtered and the volume was adjusted to 40 mL. The amount of nitrogen (N) was estimated through the Kjeldahl method as reported by Bremner [53]. The content of phosphorus (P) was assessed through the methodology outlined by Murphy and Riley [54]. The content of Cr was estimated through an atomic absorption spectrometer (AA6300C, Shimadzu, Kyoto, Japan). The levels of calcium (Ca) and potassium (K) were estimated utilizing the methodology described by Wolf [55].

### 2.8. Determination of Leaf Photosynthetic Pigment Levels

As outlined by Hiscox and Israelstam [56], extraction of fresh plant leaves’ content was performed utilizing dimethyl sulfoxide (DMSO). The absorbance was monitored at 480, 510, 645, and 663 nm to determine the levels of chlorophyll *a*, *b*, and carotenoids. DMSO served as a blank.

### 2.9. Estimation of Content of Leaf Soluble Sugar, Soluble Proteins, Proline, and Glycine Betaine

The procedure involved grinding and macerating the fresh plant leaves with 1 mL of 100 mM Tris buffer (pH of 8.0). The mixture was then exposed to centrifugation at 20,000× *g* and 6 °C for 14 min. The soluble sugar amount was quantified following the method outlined by Dey [57]. Soluble protein amount was estimated using Bradford’s method [58]. Proline level was estimated as outlined by Bates et al. [59], and the absorbance was spectrophotometrically monitored at 520 nm utilizing toluene as a blank. Additionally, the content of glycine betaine (GB) was quantified as outlined by Grieve and Grattan [60], and the absorbance was recorded at 365 nm. A control experiment was also performed by dissolving GB (50–200 mg mL^−1^) in 1 N H_2_SO_4_.

### 2.10. Determination of Leaf Gas-Exchange Properties and Relative Water Content

The net photosynthesis rate (*P_n_*), stomatal conductance (*g_s_*), and transpiration rate (*E*) were measured in fresh plant leaves in the early morning utilizing a portable gas-exchange system *LCpro+* (ADC BioScientific Ltd., Hoddesdon, UK) as per the procedure described by Holá et al. [61]. The relative water content of the leaves (LRWC) was monitored using the procedure outlined by Yamasaki and Dillenburg [62].

### 2.11. Determination of Concentration of Hydrogen Peroxide and Lipid Peroxidation in Plant Leaves

Hydrogen peroxide (H_2_O_2_) content was determined in leaves through grinding 50 mg of leafy tissue into 0.5 mL of 0.1% TCA and then centrifuging the homogenate at a high speed for 5 min. H_2_O_2_ concentration was then determined as outlined by Velikova et al. [63] and El-Esawi et al. [64]. The level of malondialdehyde (MDA) was also estimated following the methodology outlined by Rao and Sresty [65]. Contents of MDA and H_2_O_2_ were represented in μM g^−1^ FW.

### 2.12. Determination of the Contents of Total Phenol and Flavonoids in Plant Leaves

The total phenol content of leaves was determined by mixing 2 g of leaf sample with 10 mL of 80% ethanol and agitating the mixture at 70 °C for 20 min. Approximately 10 mL of distilled water containing 1 N Folin–Ciocalteau reagent (500 μL) was then added to 2 mL ethanolic extract and left to react at 30 °C. The mixture’s optical density was then recorded at 750 nm following the method of Slinkard and Singleton [66].

Total flavonoids content was estimated ustilizing the protocol outlined by Zhishen et al. [67], which involved homogenizing 1 g of powdered leaf material in 100 mL of sterile water, filtering the resulting homogenates, and mixing them with sterile water, AlCl_3_, NaNO_2,_ and NaOH. Absorbance was then monitored at 510 nm using a catechin calibration curve.

### 2.13. Estimation of Leaf Enzymatic and Non-Enzymatic Antioxidants in Plant Leaves

For enzymes assay, leafy tissues were ground in a mixture of 100 mM Tris-HCl with a pH of 7.5, 5 mM dithiothreitol, 10 mM MgCl_2_, aproptinin (1 μg.mL^−1^), 5 mM magnesium acetate, 1 mM EDTA, 1 mM phenylmethanesulfonyl fluoride, and 1.5% PVP-40. The homogenates were then exposed to filtration and centrifugation for 12 min at 10,000 rpm. The supernatant was then used for an enzyme assay. The activity of ascorbate peroxidase (APX) was assessed by extracting the leafy samples in 2 mM AsA. The superoxide dismutase (SOD) level was estimated according to the nitroblue tetrazolium (NBT) photoreduction methodology as outlined by Kono [68], and optical density was then monitored at 540 nm. Peroxidase (POD) level was estimated as outlined by Putter and Becker [69] by measuring the production rate of oxidized guaiacol spectrophotometrically at 436 nm. 

The procedures described by Nakano and Asada [70] were used to determine APX activity, where the absorbance was spectrophotometrically monitored at 265 nm. Catalase (CAT) activity was determined as described by Aebi [49], and the absorbance was monitored at 240 nm.

The level of ascorbic acid (AsA) in the plant leaves was calculated as per Mukherjee and Choudhuri [47] by extracting the fresh leaves in 6% (*w*/*v*) trichloroacetic acid, followed by mixing the resulting extract with 10% (*w*/*v*) thiourea and 2% (*w*/*v*) dinitrophenyl-hydrazine. The mixed solution was subsequently maintained in a water bath at high temperature for 20 min, cooled and then centrifuged at 1000 rpm for 10 min at 5 °C. After dissolving the obtained pellets in 80% H_2_SO_4_, absorbance was monitored at 530 nm. The level of glutathione (GSH) was also calculated as previously outlined by Yu et al. [71], and the results were represented as nM g^−1^ FW.

### 2.14. Transcriptional Analysis of Stress-Related Genes in Plant Leaves

Expression of antioxidant genes (*CAT*, *APX*, and *SOD*) and stress-associated genes (*CHS*, *DREB2A*, *CHI*, and *PAL*) was assessed in the leaves of chickpea plants inoculated with *A. brasilense* EMCC1454 under natural and Cr stress circumstances using the quantitative real-time PCR (qRT-PCR) method. In brief, isolation of the total RNA from leaf tissues was conducted by the Qiagen RNeasy Plant Mini kit, subsequently discarding the contaminating DNA and then synthesizing the cDNA first strand using the Qiagen Reverse Transcription kit. The qRT-PCR experiment was performed three times utilizing the QuantiTect SYBR Green PCR kit purchased from Qiagen. Amplification conditions were established as previously outlined by Parsottambhai [19]. The qRT-PCR reactions were carried out using earlier designed primers for the antioxidant genes [72] and the stress-associated genes [18,19,73]. Actin was assayed as a housekeeping gene, and the relative gene expression levels were calculated using 2^−ΔΔCt^ method.

### 2.15. Data Statistical Analysis

The resulting data were subjected to analysis of variance (ANOVA) as well as Duncan’s multiple range test. Data represent means ± standard error (SE) (*n* = 5), which differ significantly at *p* ≤ 0.05.

## 3. Results and Discussion

### 3.1. Growth and Chromium Tolerance of A. brasilense EMCC1454

**Figure 1:**
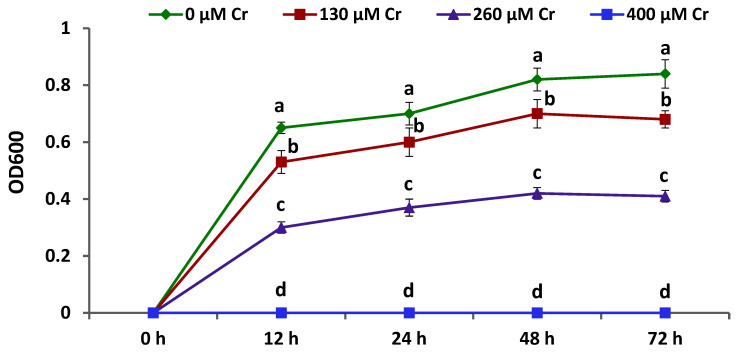
Growth curve of *A. brasilense* EMCC1454 in nutrient broth medium containing 0, 130, 260, and 400 µM K_2_Cr_2_O_7_ after 0, 12, 24, 48, and 72 h of incubation at 28 °C. OD indicates optical density. Different letters denote significant difference (*p* ≤ 0.05).

Chickpea growth and productivity are restricted by heavy metal stress, which is a main abiotic factor prevalent worldwide [34]. Nevertheless, recent reports demonstrated that PGPR application is a promising approach to boost plant yield and cope with the inhibitory effects of cadmium and arsenic stresses [34,35]. Therefore, searching for new heavy metal-tolerant bacterial isolates is of utmost importance to further enhance chickpea growth, yield, and tolerance of other major heavy metals stresses including from Cr. The current study investigated the performance and Cr tolerance of *A. brasilense* EMCC1454. The results revealed that *A. brasilense* EMCC1454 was able to thrive in nutrient broth containing 0, 130, and 260 µM K_2_Cr_2_O_7_ at different incubation periods, yielding OD = 0.65–0.84, OD = 0.53–0.70, and OD = 0.30–0.42, respectively (Figure 1). The bacterium, however, was unable to thrive in the medium containing 400 µM K_2_Cr_2_O_7_. These results indicate that *A. brasilense* EMCC1454 exhibited great capability to tolerate Cr stress up to 260 µM K_2_Cr_2_O_7_, suggesting its great potential to enhance Cr stress tolerance of food crops including chickpea.

### 3.2. Plant Growth-Promoting Characteristics of A. brasilense EMCC1454

Previous studies have demonstrated that certain soil rhizobacteria possess the capacity to form substances that can maintain plant cell turgidity, boost plant growth, and augment their resistance to stresses, including phytohormones, ACC deaminase, ammonia, hydrogen cyanide, siderophores, exopolysaccharides, and osmolytes [34,35,74,75]. In the present study, the results of PGP trait analysis showed that *A. brasilense* EMCC1454 was able to fix nitrogen, solubilize phosphate, and generate siderophores and three hydrolytic enzymes (amylase, protease, and cellulase) (Table 1 and Table 2). Such activities could help providing the plant with important, required nutrients. Siderophores, particularly, are chelating agents that can bind iron and transport it to cells through an energy dependent process to assist in chlorophyll biosynthesis and chloroplast development [76]. These results demonstrate that *A. brasilense* EMCC1454 application could be an effective way to boost the growth of plants and their resistance to environmental stresses. In agreement with our results, similar plant growth-promoting traits have been observed in other strains of *Azospirillum* species [25,26].

### 3.3. Ability of A. brasilense EMCC1454 to Generate Acid Compounds

**Figure 2:**
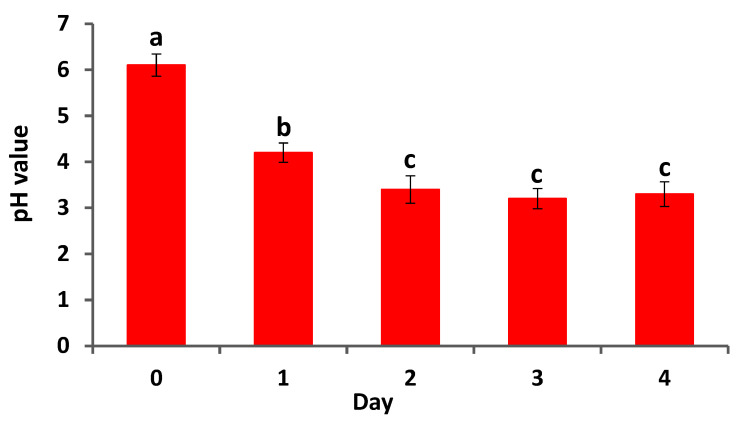
pH values of the bacterial suspension following metabolizing glucose by *A. brasilense* EMCC1454. Data represent means ± SE (*n* = 5). Different letters demonstrate significant difference (*p* ≤ 0.05).

The current study investigated the acid compound production potential of *A. brasilense* EMCC1454. The results indicated that *A. brasilense* EMCC1454 effectively lowered the pH of the bacterial culture at different incubation intervals (Figure 2), suggesting its high efficiency to form acid compounds. In a comparable investigation performed by Liu et al. (2021), it was found that two bacterial strains, namely *Lelliottia jeotgali* MR2 and *Klebsiella michiganensis* TS8, were able to boost cadmium stress tolerance by producing acid compounds and in vitro growth-promoting characteristics.

### 3.4. Impacts of Chromium Stress on PGP Traits of A. brasilense EMCC1454

**Figure 3:**
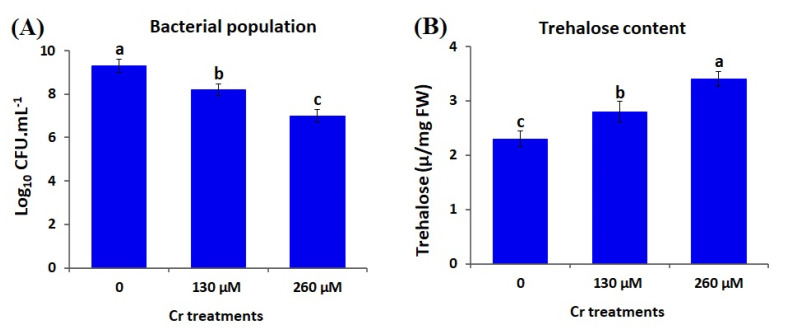
Bacterial population and trehalose content of *A. brasilense* EMCC1454 growing under Cr stress levels. Values are means ± SE (*n* = 5). Different letters demonstrate significant difference (*p* ≤ 0.05).

**Figure 4:**
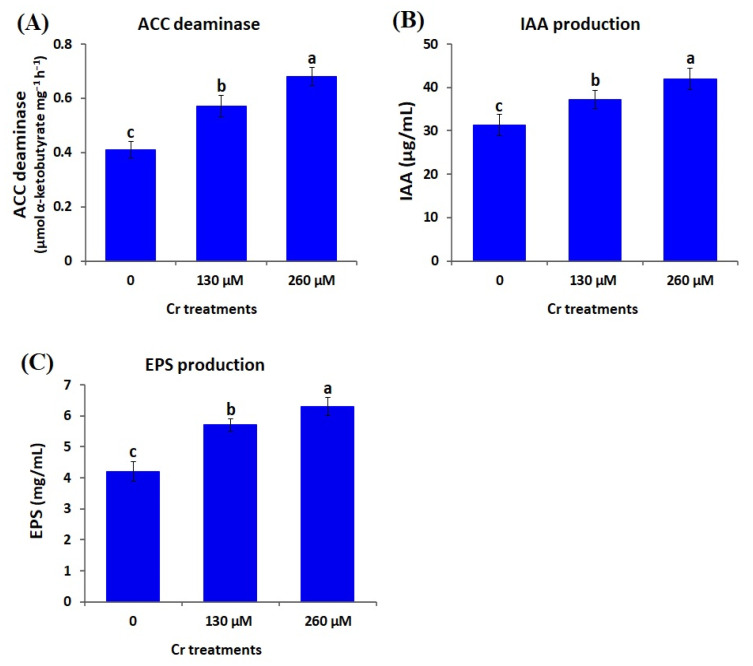
Determination of ACC deaminase, indole acetic acid (IAA), and exopolysaccharides (EPS) produced by *A. brasilense* EMCC1454 growing under Cr stress levels. Data represent means ± SE (*n* = 5). Different letters denote significant difference (*p* ≤ 0.05).

Recent reports demonstrated significant consequences of environmental stresses (salinity and heavy metals) on the stress-tolerant traits of PGPR, such as survivability, biofilms, ACC deaminase activity, minerals uptake, and generation of exopolysaccharide and IAA [34,42,77]. In the current study, the bacterial population in culture media amended with 0, 130, and 260 µM K_2_Cr_2_O_7_ was measured to determine the survivability of *A. brasilense* EMCC1454. The results revealed that the bacterium displayed optimal growth at 0 µM K_2_Cr_2_O_7_. The bacterial growth was reduced gradually by increasing Cr stress levels (Figure 3A). The results also demonstrated that the two Cr stress doses (130 and 260 µM) induced the level of trehalose, ACC deaminase, indole acetic acid, and exopolysaccharides in comparison with control conditions (0 µM) (Figure 3B and Figure 4). The highest levels of trehalose (3.4 µ/mg FW), ACC deaminase (0.68 µmol α-ketobutyrate mg^−1^ h^−1^), IAA (41.9 µg mL^−^^1^), and exopolysaccharides (6.3 mg mL^−^^1^) were recorded at 260 µM K_2_Cr_2_O_7_ (Figure 3B and Figure 4).

The levels of trehalose, ACC deaminase, IAA, and exopolysaccharides produced by *A. brasilense* EMCC1454 were higher than those generated by other *Azospirillum* isolates studied in previous reports [78,79]. The elevated levels of the crucial hormone IAA generated by *A. brasilense* EMCC1454 could potentially boost plant root growth, as well as aid in the absorption of water and nutrients [42]. Additionally, ACC serves as the building block for ethylene synthesis, and increased levels of ACC deaminase can efficiently break down ACC and alleviate stress [80]. The higher production of bacterial exopolysaccharides could also assist in reducing the negative effects of stresses through enhancing plant growth, weight and water content [42,81].

Furthermore, the ongoing research work examined the impacts of varying levels of Cr stress on the concentrations of antioxidant enzymes and total antioxidant capacity of extracts of *A. brasilense* EMCC1454. The results showed that Cr stress levels (130 and 260 µM K_2_Cr_2_O_7_) induced antioxidant enzymes levels (APX, CAT, POD, SOD) of *A. brasilense* EMCC1454 compared to control (0 µM K_2_Cr_2_O_7_) (Table 3). Among the various Cr stress levels tested, 260 µM exhibited the highest activity for APX, CAT, POD, SOD (0.11, 0.10, 0.15, and 0.08 U min^−1^ mg^−1^ protein, respectively). Moreover, the results of the current work illustrated that Cr stress levels (130 and 260 µM K_2_Cr_2_O_7_) had modulatory effects on the overall antioxidant potential of *A. brasilense* EMCC1454 extracts calculated by β-carotene-linoleic acid, DPPH, and SOS activity assays (Table 4). Overall, the induction of the antioxidant system of *A. brasilense* EMCC1454 enhanced its potential to tolerate the harmful consequences of environmental stress.

### 3.5. A. brasilense EMCC1454 Enhances Chickpea Growth and Regulates its Minerals Absorption under Chromium Stress

The current work examined the influence of *A. brasilense* EMCC1454 on growth traits and mineral absorption of chickpea plants under natural and Cr stress circumstances. The findings indicated that both Cr stress doses (130 and 260 µM K_2_Cr_2_O_7_) significantly lowered the length and dry weight of chickpea roots and shoots as well as root/shoot ratio as compared to control conditions (0 µM K_2_Cr_2_O_7_) (Table 5). The highest Cr stress level (260 µM K_2_Cr_2_O_7_) was recorded with the greatest decrease in all plant growth traits measured. On the other hand, *A. brasilense* EMCC1454 inoculation significantly increased the root length by 12.5% at T4 (130 µM of K_2_Cr_2_O_7_ + *A. brasilense* EMCC1454) and 20.1% at T6 (260 µM of K_2_Cr_2_O_7_ + *A. brasilense* EMCC1454) as compared to T3 (130 µM of K_2_Cr_2_O_7_) and T5 (260 µM of K_2_Cr_2_O_7_) treatments, respectively (Table 5). Similarly, under Cr stress conditions, *A. brasilense* EMCC1454 inoculation also induced the shoot length, dry weights of roots and shoots, and root/shoot ratio as compared to uninoculated chickpea plants (Table 5). These findings demonstrate that *A. brasilense* EMCC1454 could enhance Cr stress tolerance in chickpea by boosting the growth of plants. In line with our results, Syed et al. [34] and Adhikary et al. [35] documented that PGPR inoculation enhanced growth traits of chickpea plants exposed to high heavy metal stress conditions.

Supplying plants with essential nutrients is an important objective to enhance crop growth and yields. Results derived from the present study indicated that both Cr stress doses (130 and 260 µM K_2_Cr_2_O_7_) significantly induced Cr uptake but reduced nutrient (N, P, Ca, and K) levels in chickpea plant leaves as compared to control conditions (0 µM K_2_Cr_2_O_7_) (Table 6). Furthermore, *A. brasilense* EMCC1454 inoculation markedly augmented the levels of N, P, Ca, and K by 19.5%, 19.3%, 28.6%, and 13.6%, respectively, in plants subjected to 130 µM of K_2_Cr_2_O_7_, in comparison with the Cr-stressed non-inoculated chickpea plants. Similarly, *A. brasilense* EMCC1454 inoculation significantly boosted the contents of N, P, Ca, and K by 16.4%, 27.5%, 33.3%, and 35.3%, respectively, in plants exposed to 260 µM of K_2_Cr_2_O_7_, when compared to the Cr-stressed uninoculated chickpea plants (Table 6). Additionally, *A. brasilense* EMCC1454 inoculation significantly reduced Cr content by 28% and 35% at 130 µM of K_2_Cr_2_O_7_ and 260 µM of K_2_Cr_2_O_7_, respectively. Our findings are in concordance with earlier investigations which stated that chickpea plants inoculated with PGPR and grown under cadmium stress circumstances exhibited higher increases in N and P content, when compared to uninoculated cadmium-stressed plants [34].

### 3.6. A. Brasilense EMCC1454 Improves Photosynthetic Pigment Biosynthesis, Relative Water Content, and Gas-Exchange Traits of Chickpea under Chromium Stress

The results revealed that both Cr stress doses (130 and 260 µM K_2_Cr_2_O_7_) markedly reduced the level of chlorophyll, carotenoid, relative water content (RWC), stomatal conductance (*g_s_*), transpiration rate (*E*), and net photosynthesis rate (*P_n_*) when compared to control conditions (Table 7 and Table 8). Our results are in harmony with the earlier results of Syed et al. [34], who revealed reductions in photosynthetic pigment contents in heavy metal-stressed chickpea. The decline in pigment content might be due to ROS interference and modifications caused to thylakoid membrane components and ultrastructure of DNA and proteins [34].

On the other hand, *A. brasilense* EMCC1454 inoculation increased the photosynthetic pigments by 15.0%, 52.9%, 23.1%, and 21.4% for chlorophyll a, b, total chlorophyll, and carotenoid levels, respectively, at 130 µM of K_2_Cr_2_O_7_ and by 16.3%, 29.6%, 19.5%, and 16.3% for the same pigments respectively at 260 µM of K_2_Cr_2_O_7_, in comparison to Cr-stressed uninoculated chickpea plants (Table 7). Additionally, under Cr stress conditions, *A. brasilense* EMCC1454 inoculation boosted P_n_, E, g_s_, and RWC in chickpea plants as compared to uninoculated plants (Table 8). These findings indicate the potentiality of *A. brasilense* EMCC1454 in inducing the pigment synthesis, gas exchange traits, and relative water content of chickpea plants under Cr stress conditions. Our findings are in line with the prior results of El-Esawi et al. [25], who indicated that *A. lipoferum* FK1 inoculation augmented stress tolerance in chickpea by boosting photosynthetic pigments, leaf relative water content, and gas-exchange properties. Syed et al. [34] and Adhikary et al. [35] also demonstrated that PGPR inoculation augmented photosynthetic pigment levels in heavy metal-stressed chickpea. Moreover, *Lolium multiforum* and *Glycine max* plants treated with metal-tolerant PGPR and grown under Cd stress exhibited considerable increments in photosynthetic pigments and other growth traits [82]. Those results are in harmony with those stated by Abd_Allah et al. [83], who assumed that the enhanced pigment content of PGPR-inoculated chickpea could be due to the changes in nutrient uptake.

### 3.7. A. Brasilense EMCC1454 Increases the Levels of Osmolytes of Chickpea under Chromium Stress

Under abiotic stresses, ROS accumulate in plant cells, causing severe oxidative damage. To address the deleterious impacts of these stresses, plants stimulate their self-defense systems through enhancing the biosynthesis of osmolytes, soluble proteins, and sugars to maintain water balance and cell turgidity [84]. Moreover, under stress circumstances, PGPR application can efficiently increase the levels of osmolytes in plants to combat the stress-induced negative impacts [85]. Soluble proteins could help adjusting plant cell osmosis under stress. Furthermore, soluble sugars help maintaining the homeostasis of plant cells [86]. Taking this information into account, the concentrations of soluble proteins and sugars of inoculated and uninoculated chickpea plants cultivated under standard and Cr stress conditions were measured in the current investigation. Results revealed that Cr stress induced soluble sugar and protein concentrations by 15.7% and 13.6% at T3 (130 µM of K_2_Cr_2_O_7_) and by 66.7% and 31.8% at T5 (260 µM of K_2_Cr_2_O_7_), respectively, over those of the T1 control (Table 9). However, treatment of Cr-stressed chickpea plants with *A. brasilense* EMCC1454 considerably induced the level of soluble sugars and proteins by 30.5% and 15.1% at T4 (130 µM of K_2_Cr_2_O_7_ + *Azospirillum brasilense* EMCC1454) and 10.5% and 19.5% at T6 (260 µM of K_2_Cr_2_O_7_ + *Azospirillum brasilense* EMCC1454) over T3 and T5 treatments, respectively (Table 9). The elevated soluble proteins level under stress might be due to the stimulation of stress-responsive protein synthesis [87]. In line with our results, elevated levels of soluble proteins and sugars were indicated by Ahmad et al. [72] in chickpea plants grown under saline stress. El-Esawi et al. [1] reported increased levels of soluble proteins and sugars in heavy metal-stressed soybean plants treated with PGPR in a comparison with uninoculated stressed plants.

Proline and glycine betaine also assist in the osmoregulation process of plant cells, maintain protein structure, scavenge ROS, and enhance photosynthesis under stress conditions [88,89]. Taking this information into account, the concentrations of proline and glycine betaine of inoculated and uninoculated chickpea plants cultivated under normal and Cr stress circumstances were measured in the present study. The results showed that Cr stress significantly boosted the levels of proline and glycine betaine by 0.7-fold and 2.15-fold at T3 (130 µM of K_2_Cr_2_O_7_) and by 2.3-fold and 7.1-fold at T5 (260 µM of K_2_Cr_2_O_7_), respectively, over those of T1 control (Table 9). However, treatment of Cr-stressed chickpea plants with *A. brasilense* EMCC1454 markedly boosted proline and glycine betaine contents by 53.3% and 94.6% at T4 (130 µM of K_2_Cr_2_O_7_ + *Azospirillum brasilense* EMCC1454) and by 25.8% and 30.2% at T6 (260 µM of K_2_Cr_2_O_7_ + *Azospirillum brasilense* EMCC1454) over T3 and T5 treatments, respectively (Table 9). In agreement with our results, enhanced levels of proline and glycine betaine were reported by El-Esawi et al. [25] in PGPR-inoculated chickpea plants cultivated under saline stress. El-Esawi et al. [1] reported increased contents of proline and glycine betaine in heavy metal-stressed soybean plants inoculated with PGPR when compared to uninoculated stressed plants.

### 3.8. A. Brasilense EMCC1454 Reduces Oxidative Stress Biomarkers and Improves the Contents of Flavonoids and Phenolics of Chickpea under Chromium Stress

**Figure 5:**
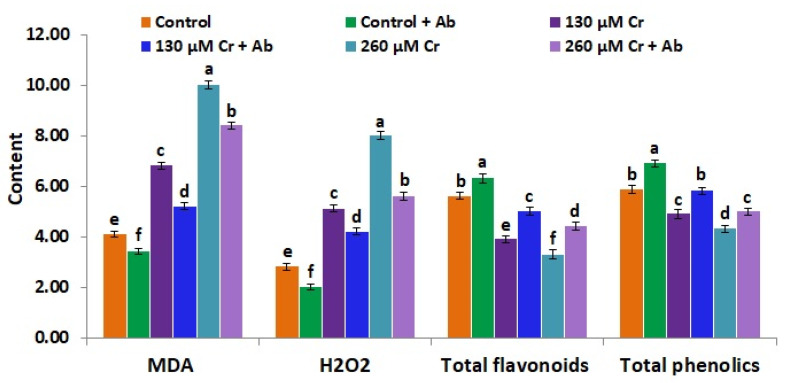
Malondialdehyde (MDA, µmol g^−1^ FW), hydrogen peroxide (µmol g^−1^ FW), total flavonoid (mg catechin/g extract), and total phenolic contents (mg gallic acid/g extract) in leaves of chickpea inoculated with *A. brasilense* EMCC1454 under natural and Cr stress conditions. Values are means ± SE (*n* = 5). Different letters denote significant difference (*p* ≤ 0.05).

Malondialdehyde (MDA) is a by-product of lipid peroxidation and refers to the stress intensity in plants [34]. Phenolics and flavonoids also assist in augmenting plant tolerance to stress. Based on that, the concentrations of MDA, H_2_O_2_, total phenolics, and total flavonoids were determined in inoculated and non-inoculated chickpea plants subjected to standard and Cr stress circumstances in the present study. Results revealed that both Cr stress doses (130 and 260 µM K_2_Cr_2_O_7_) markedly augmented the contents of MDA and H_2_O_2_ but reduced the contents of total phenolics and flavonoids in chickpea in a comparison with control (0 µM K_2_Cr_2_O_7_) (Figure 5). On the other hand, under both Cr doses, chickpea plants treated with *A. brasilense EMCC1454* had decreased levels of MDA and H_2_O_2_ and elevated contents of total phenolics and flavonoids when compared to uninoculated plants (Figure 5). These results indicate that *A. brasilense EMCC1454* could regulate the membrane function through enhancing ROS-scavenging activities. Our findings are in line with the earlier results of El-Esawi et al. [25], who revealed that *A. lipoferum* FK1 inoculation could reduce the levels of MDA and H_2_O_2_ but induce the contents of flavonoids and phenolics in salt-stressed chickpea plants. Moreover, PGPR inoculation was efficiently able to reduce MDA production in chickpea plants cultivated under heavy metal-stress circumstances [34,35]. El-Esawi et al. [1] also recorded reduced contents of MDA and H_2_O_2_ and elevated contents of total phenolics and flavonoids in heavy metal-stressed soybean plants inoculated with PGPR when compared to uninoculated stressed plants.

### 3.9. A. Brasilense EMCC1454 Induces Levels of Enzymatic and Non-Enzymatic Antioxidants of chickpea Plants under Chromium Stress

**Figure 6:**
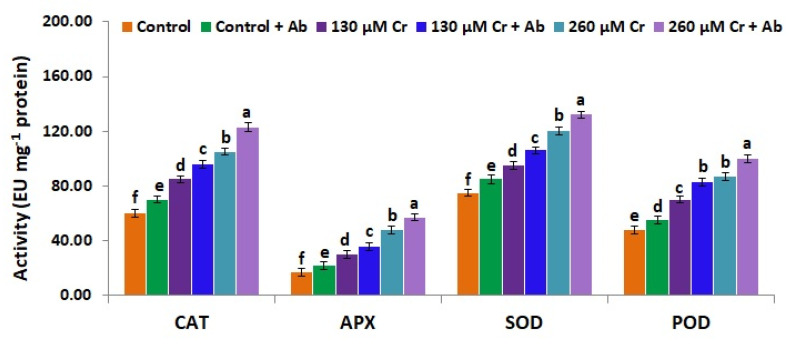
Levels of ascorbate peroxidase (APX), catalase (CAT), superoxide dismutase (SOD), and peroxidase (POD) in leaves of chickpea inoculated with *A. brasilense* EMCC1454 under natural and Cr stress conditions. Values are means ± SE (*n* = 5). Different letters denote significant difference (*p* ≤ 0.05).

**Figure 7:**
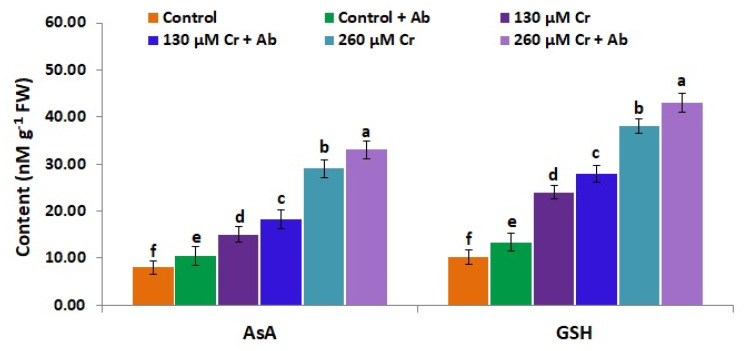
Levels of ascorbic acid (AsA) and glutathione (GSH) in leaves of chickpea inoculated with *A. brasilense* EMCC1454 under natural and Cr stress conditions. Values are means ± SE (*n* = 5). Different letters denote significant difference (*p* ≤ 0.05).

To alleviate stress toxicity and mitigate the harmful overproduction of free radicals, plants evolve diverse strategies to stimulate their ROS-scavenging antioxidant enzymes and redox components (ascorbic acid and glutathione) involved in ascorbate–glutathione cycles [25]. Taking this into account, the present investigation assessed the effects of *A. brasilense EMCC1454* on the levels of ROS-scavenging enzymes and redox components in chickpea plants exposed to normal and Cr stress circumstances. The results revealed that both Cr stress doses (130 and 260 µM K_2_Cr_2_O_7_) significantly induced the levels of the antioxidant enzymes (CAT, APX, SOD, and POD), ascorbic acid, and glutathione in chickpea plants when compared to control (0 µM K_2_Cr_2_O_7_) (Figure 6 and Figure 7). On the other hand, under both Cr doses, chickpea plants treated with *A. brasilense EMCC1454* exhibited higher levels of antioxidant enzymes, ascorbic acid, and glutathione in comparison with uninoculated plants (Figure 6 and Figure 7). The maximum values for CAT, APX, SOD, and POD activities, ascorbic acid, and glutathione levels were recorded for chickpea plants that received 260 µM of K_2_Cr_2_O_7_ and inoculated by *A. brasilense* EMCC1454 (Figure 6 and Figure 7). Therefore, *A. brasilense* EMCC1454 inoculation further improved the antioxidant defense systems under stress circumstances, causing further removal of harmful free radicals and inhibition of membrane dysfunction. These results are consistent with the earlier results of El-Esawi et al. [25] and Abd_Allah et al. [83], who reported that PGPR inoculation further induced the antioxidant enzymes and redox components in chickpea under saline stress circumstances. El-Esawi et al. [1] also reported elevated levels of antioxidant enzymes in heavy metal-stressed soybean plants treated with PGPR in comparison with uninoculated stressed plants. The elevated level of POD enzyme in *A. brasilense* EMCC1454-treated plants might be attributed to the improved Cr stress resistance mediated by the enhanced lignin synthesis [90]. The elevated CAT activity in *A. brasilense* EMCC1454-inoculated chickpea plants subjected to Cr stress could be attributed to the reduced level of H_2_O_2_ in the apoplast [91]. In addition, elevated levels of SOD, APX, CAT, and POD in PGPR-treated chickpea plants under Cr stress could induce plant growth through the protection of chloroplasts and other organelles where the essential metabolic processes happen [92]. Moreover, *A. brasilense* EMCC1454-induced generation of redox components under control and Cr stress conditions might also be attributed to the photosynthetic electron transport chain maintenance, resulting in a decrease in free radical formation [93].

### 3.10. A. Brasilense EMCC1454 Induces the Expression of Antioxidant Genes and Genes Mediating Tolerance to Chromium Toxicity

**Figure 8:**
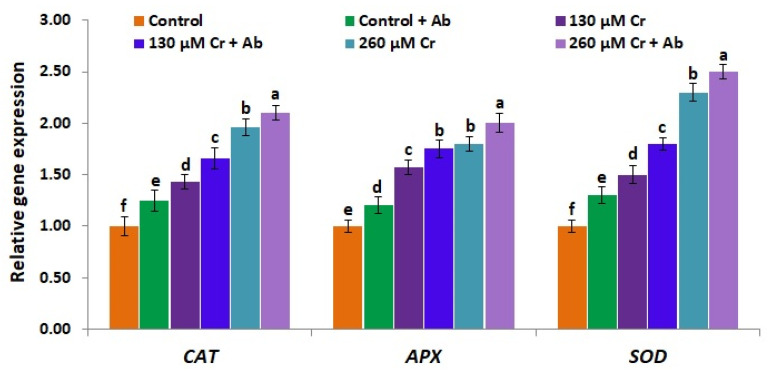
Expression level of ROS-scavenging (*CAT*, *APX* and *SOD*) genes in leaves of chickpea inoculated with *A. brasilense* EMCC1454 under natural and Cr stress conditions. Values are means ± SE (*n* = 5). Different letters denote significant difference (*p* ≤ 0.05).

**Figure 9:**
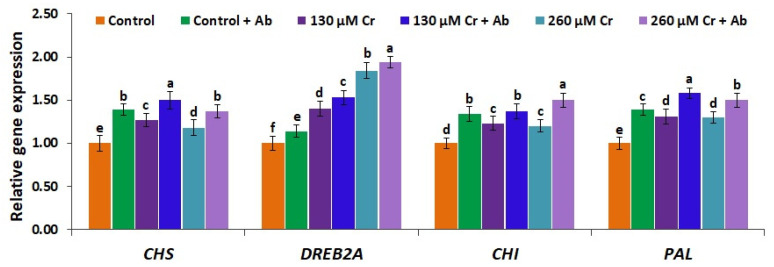
Expression level of *CHS*, *DREB2A*, *CHI,* and *PAL* genes in leaves of chickpea inoculated with *A. brasilense* EMCC1454 under natural and Cr stress conditions. Values are means ± SE (*n* = 5). Different letters denote significant difference (*p* ≤ 0.05).

In consistence with the Cr-induced higher levels of antioxidant enzymes in chickpea plants, the expression levels of ROS-scavenging genes (*CAT*, *SOD,* and *APX*) as well as genes conferring resistance to Cr stress (*CHS*, *DREB2A*, *CHI,* and *PAL*) were also significantly upregulated in chickpea plants subjected to the two Cr stress doses (130 and 260 µM K_2_Cr_2_O_7_) when compared to the non-stressed plants (0 µM K_2_Cr_2_O_7_) (Figure 8 and Figure 9). Similarly, previous studies reported the induced expression of ROS-scavenging genes and other stress tolerance-associated genes in plants subjected to stress circumstances [94,95]. Moreover, *A. brasilense* EMCC1454 inoculation further induced the expression of ROS-scavenging genes as well as Cr-tolerance genes in chickpea subjected to two Cr stress doses (130 and 260 µM K_2_Cr_2_O_7_) in comparison with plants subjected to Cr stress alone (Figure 8 and Figure 9). The maximum expression levels of all genes were recorded for chickpea plants that received 260 µM of K_2_Cr_2_O_7_ and were inoculated by *A. brasilense* EMCC1454 (Figure 8 and Figure 9). These higher expression levels might be due to the interactions of *A. brasilense* EMCC1454 with chickpea plants and thus inducing the production of useful substances such as phytohormones and siderophore. These substances could in turn help reducing ROS formation through regulating the solutes and antioxidant biosynthesis pathways, leading to improved Cr tolerance in plants. Our results are in line with the prior findings of El-Esawi et al. [25], who recorded significantly elevated expression levels of stress-tolerant genes in PGPR-inoculated chickpea plants under stress circumstances. Other studies also reported higher expression levels of antioxidant genes and heavy metal stress-tolerant genes in PGPR-inoculated soybean plants grown under heavy metal stress [1]. PGPR inoculation alleviated the salt-induced damage in wheat plants through inducing the expression levels of crucial transport proteins involved in compartmentalization of toxic ions [96].

## 4. Conclusions

The present study revealed that Cr stress has inhibitory effects on the growth traits, nutrients acquisition, and pigment content of chickpea plants, thereby modulating their physiobiochemical and molecular mechanisms. Contrarily, *A. brasilense* EMCC1454 inoculation proved to be an effective approach to boost chickpea growth and mitigate the adverse impact of Cr toxicity on chickpea plants through interacting with the plant and stimulating the production of phytohormones and beneficial PGP substances. *A. brasilense* EMCC1454 inoculation significantly enhanced the photosynthesis machinery, osmolyte formation, and antioxidants levels. Furthermore, this bacterial inoculation markedly upregulated the expression of ROS-scavenging (*CAT*, *APX,* and *SOD*) genes along with the genes conferring Cr stress tolerance (*CHS*, *DREB2A*, *CHI,* and *PAL*), causing significant reductions in ROS formation in chickpea plants. Taken together, the results of the current study demonstrated the promising role of *A. brasilense* EMCC1454 in enhancing chickpea growth and alleviating the adverse impacts of Cr toxicity in chickpea plants grown under Cr stress.

## Figures and Tables

**Table 1:** Nitrogen fixation, siderophore formation, and phosphate solubilization of the bacterium *A. brasilense* EMCC1454.

Nitrogen Fixation	Siderophore Formation	Phosphate Solubilization
+	+	+

“+” sign represents the presence of a trait.

**Table 2:** Hydrolytic enzymes activities of the bacterium *A. brasilense* EMCC1454.

Amylase	Protease	Chitinase	Cellulase
+	+	−	+

“+” sign represents the presence of a trait, while “−” represents the absence of a trait.

**Table 3:** Levels of antioxidant enzymes (U min^−1^ mg^−1^ protein) of the bacterium *A. brasilense* EMCC1454 subjected to Cr stress.

Cr (µM)	APX	CAT	POD	SOD
0	0.06 ± 0.02 c	0.04 ± 0.01 c	0.08 ± 0.02 c	0.03 ± 0.01 c
130	0.08 ± 0.02 b	0.07 ± 0.02 b	0.11 ± 0.02 b	0.06 ± 0.02 b
260	0.11 ± 0.03 a	0.10 ± 0.02 a	0.15 ± 0.03 a	0.08 ± 0.02 a

Data are mean values ± standard error (*n* = 5). Values followed by different letters differ significantly (*p* ≤ 0.05).

**Table 4:** Total antioxidant capacity of the extracts of *A. brasilense* EMCC1454 subjected to normal and Cr stress conditions.

Cr (µM)	β-Carotene-Linoleic Acid (IC_50_, μg mL^−1^)	DPPH (IC_50_, μg mL^−1^)	Superoxide-Scavenging (SOS) Activity (%)
0	0.46 ± 0.05 a	0.41 ± 0.03 a	32.44 ± 1.32 a
130	0.42 ± 0.04 b	0.37 ± 0.04 b	27.61 ± 1.22 b
260	0.37 ± 0.04 c	0.32 ± 0.04 c	23.48 ± 1.20 c

Data are mean values ± standard error (*n* = 5). Values followed by different letters differ significantly (*p* ≤ 0.05).

**Table 5:** Morphological parameters of chickpea plants treated with *A. brasilense* EMCC1454 under natural and Cr stress conditions.

Cr (µM)	*A. Brasilense* EMCC1454	Root Length (cm plant^−1^)	Shoot Length (cm plant^−1^)	Root Dry Weight (g plant^−1^)	Shoot Dry Weight (g plant^−1^)	Root DW/Shoot DW
0	−Ab (T1)	21.32 ± 1.67 b	35.67 ± 1.78 b	8.03 ± 0.34 b	8.91 ± 0.37 b	0.90 ± 0.06 b
	+Ab (T2)	22.99 ± 1.43 a	37.96 ± 1.43 a	9.11 ± 0.29 a	9.86 ± 0.31 a	0.92 ± 0.09 a
130	−Ab (T3)	17.01 ± 1.28 d	30.12 ± 1.82 d	5.98 ± 0.22 d	6.77 ± 0.28 d	0.88 ± 0.06 c
	+Ab (T4)	19.13 ± 1.32 c	33.25 ± 1.92 c	6.97 ± 0.27 c	7.72 ± 0.25 c	0.90 ± 0.09 b
260	−Ab (T5)	10.81± 1.17 f	24.82 ± 1.53 f	3.59 ± 0.31 f	5.02 ± 0.24 f	0.72 ± 0.09 e
	+Ab (T6)	12.98± 1.11 e	28.11 ± 1.61 e	5.01 ± 0.33 e	6.13 ± 0.32 e	0.82 ± 0.07 d

−Ab, absence of *A. brasilense* EMCC1454 inoculation; +Ab, presence of *A. brasilense* EMCC1454 inoculation; DW, dry weight. Data are means ± standard error (*n* = 5). Different letters show significant differences (*p* ≤ 0.05).

**Table 6:** Analysis of elements (mg g^−1^ DW) in leaves of chickpea plants treated with *A. brasilense* EMCC1454 under natural and Cr stress conditions.

Cr (µM)	*A. Brasilense* EMCC1454	Cr	N	P	Ca	K
0	−Ab (T1)	0.02 ± 0.01 d	29.2 ± 0.10 b	19.9 ± 0.93 b	2.0 ± 0.09 b	10.2 ± 0.31 b
	+Ab (T2)	0.01 ± 0.01 d	32.8 ± 0.13 a	21.9 ± 0.81 a	2.3 ± 0.08 a	11.6 ± 0.28 a
130	−Ab (T3)	0.25 ± 0.02 b	24.1 ± 0.11 c	15.0 ± 0.94 d	1.4 ± 0.06 d	8.1 ± 0.25 d
	+Ab (T4)	0.18 ± 0.01 c	28.8 ± 0.12 b	17.9 ± 0.93 c	1.8 ± 0.09 c	9.2 ± 0.22 c
260	−Ab (T5)	0.40 ± 0.01 a	17.7 ± 0.14 e	10.2 ± 0.88 f	0.9 ± 0.07 f	5.1 ± 0.21 f
	+Ab (T6)	0.26 ± 0.02 b	20.6 ± 0.15 d	13.0 ± 0.96 e	1.2 ± 0.09 e	6.9 ± 0.24 e

−Ab, absence of *A. brasilense* EMCC1454 inoculation; +Ab, presence of *A. brasilense* EMCC1454 inoculation; DW, dry weight. Data are means ± standard error (*n* = 5). Different letters show significant differences (*p* ≤ 0.05).

**Table 7:** Contents of photosynthetic pigments (mg g^−1^ FW) in leaves of chickpea plants treated with *A. brasilense* EMCC1454 under natural and Cr stress conditions.

Cr (µM)	*A. Brasilense* EMCC1454	Chlorophyll *a*	Chlorophyll *b*	Total Chlorophyll	Carotenoid
0	−Ab (T1)	1.44 ± 0.06 b	0.53 ± 0.05 b	1.97 ± 0.07 b	0.36 ± 0.04 c
	+Ab (T2)	1.61 ± 0.05 a	0.60 ± 0.03 a	2.21 ± 0.09 a	0.43 ± 0.06 b
130	−Ab (T3)	1.13 ± 0.06 d	0.34 ± 0.04 c	1.47 ± 0.05 d	0.42 ± 0.04 b
	+Ab (T4)	1.30 ± 0.04 c	0.52 ± 0.04 b	1.81 ± 0.08 c	0.51 ± 0.05 a
260	−Ab (T5)	0.86 ± 0.05 f	0.27 ± 0.03 d	1.13 ± 0.06 f	0.43 ± 0.04 b
	+Ab (T6)	1.00 ± 0.05 e	0.35 ± 0.05 c	1.35 ± 0.08 e	0.50 ± 0.06 a

−Ab, absence of *A. brasilense* EMCC1454 inoculation; +Ab, presence of *A. brasilense* EMCC1454 inoculation; FW, fresh weight. Data are means ± standard error (*n* = 5). Different letters show significant differences (*p* ≤ 0.05).

**Table 8:** Gas-exchange properties and relative water content in leaves of chickpea plants inoculated with *A. brasilense* EMCC1454 under natural and Cr stress conditions.

Cr (µM)	*A. Brasilense* EMCC1454	*P_n_* (μmol m^−2^ s^−1^)	*E* (mmol m^−2^ s^−1^)	*G_s_* (mol m^−2^ s^−1^)	RWC (%)
0	−Ab (T1)	10.03 ± 0.39 b	1.65 ± 0.11 b	0.10 ± 0.02 b	82.7 ± 2.91 b
	+Ab (T2)	11.10 ± 0.42 a	1.74 ± 0.10 a	0.12 ± 0.02 a	85.1 ± 2.93 a
130	−Ab (T3)	8.01 ± 0.38 c	1.41 ± 0.11 d	0.06 ± 0.01 d	64.4 ± 2.82 d
	+Ab (T4)	9.94 ± 0.41 b	1.52 ± 0.10 c	0.08 ± 0.02 c	71.6 ± 2.77 c
260	−Ab (T5)	5.96 ± 0.30 e	1.17 ± 0.10 f	0.04 ± 0.02 e	49.2 ± 2.91 e
	+Ab (T6)	7.02 ± 0.37 d	1.28 ± 0.11 e	0.06 ± 0.03 d	63.9 ± 2.96 d

−Ab, absence of *A. brasilense* EMCC1454 inoculation; +Ab, presence of *A. brasilense* EMCC1454 inoculation. Data represent means ± standard error (*n* = 5). Different letters show significant differences (*p* ≤ 0.05).

**Table 9:** Levels of soluble sugar, protein, proline, and glycine betaine in leaves of chickpea plants inoculated with *A. brasilense* EMCC1454 under natural and Cr stress conditions.

Cr (µM)	*A. Brasilense* EMCC1454	Soluble Sugars (mg g^−1^ FW)	Proteins (mg g^−1^ of FW)	Proline (µg g^−1^ FW)	Glycine Betaine (µmol g^−1^ FW)
0	−Ab (T1)	5.98 ± 0.57 e	19.8 ± 1.32 e	26.2 ± 1.88 f	3.07 ± 0.22 f
	+Ab (T2)	6.81 ± 0.46 d	20.8 ± 1.42 d	33.3 ± 1.89 e	5.63 ± 0.31 e
130	−Ab (T3)	6.92 ± 0.62 d	22.5 ± 1.38 c	44.5 ± 1.82 d	9.68 ± 0.76 d
	+Ab (T4)	9.03 ± 0.59 c	25.9 ± 1.53 b	68.2 ± 1.94 c	18.84 ± 1.02 c
260	−Ab (T5)	9.97 ± 0.66 b	26.1 ± 1.44 b	86.6 ± 3.32 b	24.86 ± 1.41 b
	+Ab (T6)	11.02 ± 0.58 a	31.2 ± 1.52 a	108.9 ± 3.83 a	32.37 ± 1.52 a

−Ab, absence of *A. brasilense* EMCC1454 inoculation; +Ab, presence of *A. brasilense* EMCC1454 inoculation; FW, fresh weight. Data are means ± standard error (*n* = 5). Different letters show significant differences (*p* ≤ 0.05).

## Data Availability

All the data supporting the findings of this study are already included in this published manuscript.
